# Long-Term Evaluation of Percutaneous Groin Access for EVAR

**DOI:** 10.1007/s00270-018-2072-3

**Published:** 2018-10-04

**Authors:** Krit Dwivedi, John Mark Regi, Trevor J. Cleveland, Douglas Turner, Dan Kusuma, Steven M. Thomas, Stephen D. Goode

**Affiliations:** Sheffield Vascular Institute, Northern General Hospital, Herries Road, Sheffield, S5 7AU UK

**Keywords:** PEVAR, Percutaneous, Proglide, Long term complications, Arterial access, Pseudoaneurysms

## Abstract

**Background:**

Percutaneous endovascular aneurysm repair (PEVAR) has been shown to have high success rates, shorter operating times and length of stay compared to open access. However, there exists a lack of long-term follow-up data on these patients, and questions remain regarding longer-term outcomes. This study aims to assess the long-term complications and evolution of accessed vessels post-PEVAR.

**Methods:**

Sixty-one cases of bilateral PEVAR (122 groins) with > 36 months follow-up were analysed. Vessel diameter, calcification, dissection, lymphocele, pseudoaneurysm and thrombus formation were reviewed at 30th day and at the most recent follow-up CT. Notes were reviewed for groin infections, haematomas and nerve injury. Complications were considered ‘major’ if they required intervention or treatment.

**Results:**

Mean follow-up time from procedure to most recent scan was 49.9 months. There were no major short- or long-term complications. The early complication rate was 9.8%, with six pseudoaneurysms, four dissections, one thrombus, one nerve injury and no lymphoceles, haematomas or groin infections. The long-term complication rate was 0.8%, with only one pseudoaneurysm. The remainder of early complications resolved naturally without intervention. Accessed vessel showed significantly (*P* ≤ 0.05) increased diameter and calcification between 30th day and last follow-up scan.

**Conclusion:**

This study provides the largest clinical cohort and the longest mean follow-up time reported in the literature and demonstrates the long-term safety of PEVAR. PEVAR has a very low long-term complication rate, without any major complications in our cohort. The accessed common femoral arteries do not show stenosis or thrombosis. Minor short-term complications appear to gradually resolve without intervention. Larger multi-centre studies are recommended.

## Introduction

EVAR is now the most common technique for repair of abdominal aortic aneurysms (AAA) in the Western world [1], due to low operative mortality, morbidity and quicker recovery compared to open repair [2]. EVAR has traditionally been performed by open surgical femoral artery access to enable the delivery of large stent graft delivery systems. Although surgical access is considered a minor procedure, access-related complications have been shown to occur in 14–22% of patients [3, 4]. With the advent of suture-mediated closure devices (SMCDs) [5], percutaneous femoral access techniques are being increasingly utilised for entirely percutaneous endovascular aneurysm repair (PEVAR) [6]. Several studies have shown PEVAR to have both a shorter operating time and shorter length of stay compared to open surgical access [4, 7]. Technical success rates vary between 86 and 94% [8–11].

Complications of PEVAR can be classified as immediate, short term or long term. The most common immediate complication is bleeding from access vessels as a result of arterial damage related to passage of the access sheath or closure device systems. Short-term complications of groin access include groin infections, nerve injury, pseudoaneurysms and arterial stenosis, occlusion, thrombosis or dissection [8, 10]. Whilst studies have reaffirmed the advantages of PEVAR over open surgical access with regard to immediate- and short-term complications [12], there exists very little data on the long-term outcomes of PEVAR groin access. There is a paucity of follow-up data looking at both the evolution of identified short-term complications and evolution of the accessed vessels. The impact of SMCDs on vessel diameter, calcification or thrombosis is unclear. A recent review by De Souza et al. found only three studies with a follow-up time greater than 2 years [13–16].

This study uniquely aims to assess the long-term outcomes of PEVAR, using a cohort of patients with mean follow-up greater than 36 months from procedure to latest scan. Its outcomes are:

1. Rate of early (30 day) and late (> 36 months) complications.
2. Evolution of accessed common femoral artery (CFA) vessel diameter.
3. Evolution of accessed CFA wall calcification.

## Methods

A retrospective cohort study was performed on all patients who underwent bilateral PEVAR at our centre between June 2009 and June 2015, providing six years of consecutive data. Bilateral PEVAR is defined as EVAR in which both access sites were managed entirely percutaneously using SMCDs. This formed the inclusion criteria and included both emergency and elective cases.

All elective cases are discussed in a multidisciplinary team (MDT) meeting with interventional vascular radiologists and vascular surgeons to discuss suitability for an entirely percutaneous approach. Factors that could potentially exclude a percutaneous approach in our clinical practice are the relative contraindications of closure devices—circumferential or a large amount of CFA anterior wall calcification, skin to vessel distance > 70 mm, obesity (BMI > 30), previous CFA cut-down, surgery or use of closure or collagen plug device in last 90 days. The clinical cohort is therefore comprised of patients who were deemed suitable for bilateral percutaneous access. Therefore, the exclusion criteria for this study are:

- Concomitant intervention at time of PEVAR.
- Lack of follow-up CT imaging.
- Cases deemed inappropriate at MDT for percutaneous access, as per the relative contradictions for percutaneous access outlined above.

Cases with a unilateral percutaneous access failure were excluded from the primary analysis of long-term evaluation of percutaneous access.

Post-PEVAR, all patients undergo a post-operative CT angiogram (CTA) at 30 days and are clinically reviewed. The results are routinely discussed in MDT. They are followed up indefinitely with annual CTA for the first 5 years and non-contrast scans after 5 years.

Data were obtained from both electronic records and case notes. Demographic information collected included age, sex and date of birth. Procedure data collected included closure device used, groin accessed, aneurysm diameter, neck length and diameter. Patient notes at clinical follow-up were reviewed for evidence of any groin infections or nerve injury from access. For each patient, the post-operative scan (usually 30 days post-procedure) and last (most recent) available follow-up scan were reviewed. CT images were reviewed by three radiologists—one trainee and two experienced consultant interventional radiologists.

From CT imaging, for each groin, CFA vessel depth and extent of calcification were measured. For the extent of calcification, a 0–3 grading system was used, as previously described by Rijkée et al. and used by Manunga et al. [14, 17]. Those with no calcification were graded ‘0’. Those with scattered posterior calcification were graded ‘1’ if < 33% (mild) or ‘2’ if between 33 and 66% (moderate) calcification. Any anterior wall calcification or posterior calcification > 66% was graded ‘3’ (severe). In addition, images were reviewed for evidence of complications of groin access, namely pseudoaneurysm or lymphocele formation, dissection or thrombosis. Complications were considered ‘major’ if they required further treatment or intervention.

### Procedural Details

All procedures were carried out in the interventional radiology suite, with anaesthetic support as required. Patients’ CFAs were assessed by the pre-procedure CT to confirm suitability for percutaneous access and checked by ultrasound assessment on the day of procedure. The puncture was made into both CFAs under direct ultrasound guidance. The suture-mediated closure device of choice was then introduced. The closure device choice was primary operator dependent. This included predominantly use of a double-Proglide/Perclose technique (two single-suture devices) and to a lesser extent Prostar XL device (single two-suture device). Procedural technical success was defined as successful CFA closure without the need for adjunct surgical or endovascular procedures.

### Statistical Analysis

Data were collected and analysed using a combination of Excel (Version 2010, Microsoft, Redmond) and SPSS (Version 23, IBM Corp, Armonk, NY). Descriptive statistics were reported as number (percentage) or mean (standard deviation), as appropriate. To assess significance of difference between paired values of vessel diameter, paired *T* test was used. The Wilcoxon matched pairs signed-rank test was used to assess significance between the ordinal groups of vessel calcification.

## Results

**Fig. 1:**
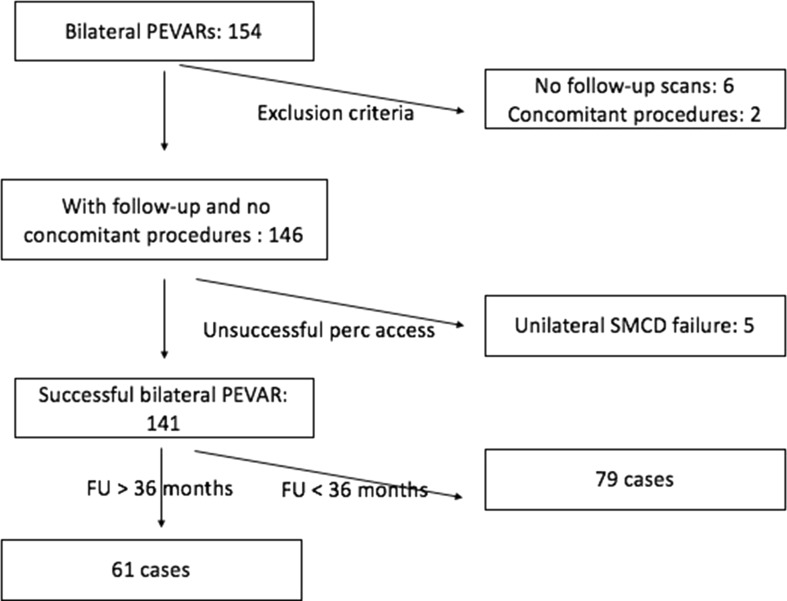
Study cohort selection with including and exclusion criteria

In the chosen six-year period, 154 bilateral PEVAR cases were performed. Eight cases were excluded: six due to the absence of follow-up CT scans, two due to concomitant procedures—one right lower pole renal embolization and one portal vein embolization. Of the remaining 146 cases, there were five cases of unilateral closure device failure, necessitating open surgical groin closure. These were excluded as the cases did not undergo bilateral percutaneous access. The technical success rate was 96.6%. Of the remaining 141 patients, 61 had follow-up data for longer than 36 months. Therefore, the study analysis cohort comprised of 61 patients and 122 groins successfully treated entirely percutaneously. Figure 1 outlines the selection of our study cohort.

Mean patient age was 75.46 ± 6.63 years, with 88.5% of patients being male. Mean follow-up time between procedure and latest follow-up scan was 49.9 ± 11.1 months, with a range of 36–76 months. Fifty-seven cases (93.4%) were performed as elective and four (6.6%) were performed as emergency. Median main body diameter was 18 French. Mean aneurysm diameter was 62.5 ± 9.7 mm, mean neck diameter was 23.0 ± 3.4 mm and mean neck length was 33.0 ± 12.7 mm. Perclose/Proglide was used in 114 (93.4%) groins, and Prostar XL was used in 8 (6.6%) groins (Tables 1 and 2).

**Table 1:** Patient and aneurysm characteristics

	Total (*n* = 61)
*Patient characteristics*	
Age at procedure (years) (mean ± SD)	75.46 ± 6.63
Follow-up time (months) (mean ± SD)	49.9 ± 11.1
Male, *n* (%)	54 (88.5%)
Female, *n* (%)	7 (11.5%)
Elective cases, *n* (%)	57 (93.4%)
Emergency cases, *n* (%)	4 (6.6%)
*Aneurysm characteristics*	
Max diameter (mm)	61.95 ± 9.12
Neck diameter (mm)	23.46 ± 3.69
Neck length (mm)	32.47 ± 12.82
Median French diameter	18 French

**Table 2:** Vessel characteristics and complications at 30-day scan and most recent follow-up scan

Groin variables	30-day scan (*n* = 122)	Most recent scan (*n* = 122)
Vessel characteristics		
Vessel diameter (mm)	10.5 ± 1.65	10.9 ± 2.1
Calcification		
0—no calcification	15 (12.3%)	3 (2.5%)
1— < 33% posterior calcification	74 (60.7%)	67 (54.9%)
2—33 to 66% posterior calcification	19 (15.6%)	22 (18%)
3— > 66% posterior or anterior calcification	14 (11.5%)	30 (24.6%)
Complications		
Pseudoaneurysms	6	1
Lymphoceles	0	0
Thrombus	1	0
Dissections	4	0

### Complications

At the first post-procedure scan, there were six pseudoaneurysms, four dissections and one case of non-occlusive thrombus identified. There were no lymphoceles. None of the complications were deemed clinically significant and were managed conservatively. Therefore, there were no major complications. At the clinic review, there was only one case of nerve injury, with no groin infections noted. Therefore, the overall ‘early’ complication rate was 9.8%.

At the latest follow-up scan, there was only one pseudoaneurysm. This changed in diameter from 7 to 5 mm and remained clinically insignificant. There were no dissections or thrombus. Therefore, the overall ‘long-term’ complication rate was 0.8%.

### CFA Vessel Diameter

At the first post-procedure scan, mean vessel diameter was 10.5 ± 1.65 cm. At the latest follow-up scan, mean vessel diameter was 10.9 ± 2.1 cm. The mean difference was statistically significant (*P* < 0.05) at 0.420 mm with a confidence interval between − 0.79 and − 0.6 mm.

### CFA Calcification

At the first post-procedure scan, 15 (12.3%) groins showed no calcification, 74 (60.7%) mild posterior wall calcification, 19 (15.6%) moderate posterior calcification and 14 (11.5%) showed severe posterior or anterior wall calcification.

At the latest follow-up scan, 3 (2.5%) groins showed no calcification, 67 (54.9%) mild posterior calcification, 22 (18%) moderate posterior calcification and 30 (24.6%) showed severe posterior or anterior calcification.

There is statistically significant (< 0.05) difference between these two paired groups using the Wilcoxon signed-rank test, with an increase in calcification in the longer follow-up group.

## Discussion

Studies investigating PEVAR outcomes have focused on short-term variables, such as technical success, length of stay, immediate- or short-term complications. This study aims to investigate longer-term outcomes, looking at a cohort of patients with > 36 months follow-up. The natural progression and evolution of accessed vessels and related complications (vessel stenosis, occlusion, thrombosis or dissection) in the long-term are currently not well understood. A recent Cochrane review noted moderate-quality evidence with no difference between the percutaneous approaches compared with formal open femoral artery access group for short-term mortality, aneurysm exclusion, major complications and wound infection [7]. However, ‘long-term’ was defined as 6 months, which highlights the paucity of evidence following up patients who have undergone PEVAR.

To our knowledge, the study with the longest mean follow-up time in the literature is by Bent et al. with 50 ± 8 months. However, it is limited to only 29 groins and a specific closure device. The authors also recognised their strict selection criteria and young cohort of patients as a contributing factor to the relatively low complication rate. Our study provides a more representative and larger clinical cohort, with 122 groins and both the major closure device strategies in a large tertiary referral centre.

### Complications

The low rate of short-term complications (9.8%) is compatible with those reported in the literature, with a recent meta-analysis reporting an overall complication rate of 7.8%. Reported pseudoaneurysm rates vary from 0 with suture-mediated closure devices to 8.5% with fascia closure techniques [16]. None of these complications were classed as ‘major’ as they were not clinically significant and did not require any further treatment or intervention.

Of note in our study is the observation that identified minor complications, when followed up, appear to resolve without intervention. Of the six pseudoaneurysms, four dissections and one thrombus, only one pseudoaneurysm remained on long-term follow-up. This remained clinically insignificant. Therefore, there were no long-term ‘major’ complications identified with percutaneous groin access. To our knowledge, this observation has not been demonstrated in any prior literature and has implications for management of the more common minor short-term complications.

### CFA Vessel Diameter

There was a slight, but statistically significant, increase in vessel diameter between the paired post-op and long-term scans. This is an important finding as it demonstrates patency of the accessed vessel in the long-term and absence of a stenotic or occlusive process. This is an important finding that refutes the notion of vessel stenosis secondary to the use of a closure device. Lee et al. [18] investigating Proglide devices believed this was due to the way the device replicates conventional surgical interrupted simple full-thickness sutures. We theorise that the most likely cause for the apparent dilatation is the underlying aneurysmal disease process in this cohort of patients, given their age and long follow-up time.

### CFA Calcification

There was a significant increase in level of calcification between post-op and long-term follow-up scans. This could be secondary to intervention, use of closure devices or due to the high natural atherosclerotic process evident in this cohort of patients secondary to ageing. There is a lack of evidence in the literature investigating the impact of vessel intervention, the use of closure devices or indeed open surgical closure on vessel calcification.

## Limitations

The retrospective nature of the study has built-in selection bias. All clinical cases were reviewed in MDT beforehand to assess suitability for bilateral PEVAR. The high rate of all-cause mortality and morbidity in this cohort of patients limits large numbers of cases with long-term follow-up.

## Conclusion

This study provides the largest clinical cohort and the longest mean follow-up time in the literature looking at long-term follow-up post-PEVAR. PEVAR has a very low complication rate in the long-term, and accessed vessels do not show evidence of stenosis or thrombosis. Clinically insignificant minor short-term complications appear to gradually resolve without intervention. This study demonstrates no major long-term complications and demonstrates the safety of PEVAR. Larger, multi-centre studies are recommended to further reaffirm these findings across different operators.
